# How effective are clinical pre-farrowing risk assessment and the use of soft rubber mats in preventing shoulder ulcers in at-risk sows?

**DOI:** 10.1186/s40813-019-0123-z

**Published:** 2019-07-12

**Authors:** Daniel Meyer, Charlotte Vogel, Lothar Kreienbrock, Elisabeth große Beilage

**Affiliations:** 1 Field Station for Epidemiology, University of Veterinary Medicine Hanover, Foundation, Buescheler Str. 9, 49456 Bakum, Germany; 2 Institute for Biometry, Epidemiology and Information Processing (IBEI), University of Veterinary Medicine Hanover, Foundation, Buenteweg 2, 30559 Hanover, Germany

**Keywords:** Scar tissue, Shoulder ulcer, Risk assessment, Rubber mat

## Abstract

**Background:**

Lameness, low BCS and scar tissue in the skin covering the tuber spina scapulae are known as risk factors for shoulder ulcer in sows. In a two-step study, the predictive ability of pre-farrowing clinical examination and the preventive effect of rubber mats on the development of shoulder ulcers in at-risk sows were evaluated.

**Material and methods:**

The study included 659 sows that were clinically examined one week before farrowing to distinguish risk sows from no-risk sows. Sows with a BCS ≤ 2 and/or a locomotion score > 3 and/or scar tissue in the skin covering the tuber spina scapulae were classified as at risk of developing shoulder ulcers. The at-risk sows were randomly assigned to either a prevention group in which sows were stalled in farrowing crates equipped with rubber mats, or a non-prevention group in which sows were stalled in standard crates. The shoulder areas were photographed during the first two weeks of the lactation period.

**Results:**

The chance of developing a shoulder ulcer was significantly higher for at-risk sows than for non-at-risk sows (OR 5.55, *p* < 0.0001). At-risk sows stalled in crates equipped with rubber mats as preventive substrates had a significantly lower chance of developing shoulder ulcers than did those stalled in standard pens (OR 0.54, *p* = 0.0358).

**Conclusions:**

The development of shoulder ulcers in sows can be predicted by clinical pre-farrowing risk assessment based on BCS, locomotion score and scar tissue scoring. Providing at-risk sows with farrowing crates equipped with rubber mats had a statistically significant protective effect.

## Background

Shoulder ulcers are frequently seen in sows that have recently farrowed [1]. The lesions typically develop in the area over the tuber spina scapulae. During farrowing, sows often lay for long periods in the same posture, which leads to the compression of blood vessels, insufficient blood circulation, necrosis and subsequent ulceration [2]. According to the tissues involved, four stages of shoulder ulcers are distinguished. Lesions that affect only the epidermis (stage 1) are differentiated from those that also affect the dermis (stage 2) or that affect all skin layers, including the subcutaneous tissue (stage 3). In stage 4, all skin layers and the underlying bone of the tuber spina scapulae are involved. Stages 3 and 4 are considered substantial animal welfare-related lesions [1].

### Risk factors

Risk factors for the development of shoulder ulcers have been identified in several studies [2–14]. Animal-related risk factors are distinguished from those associated with the environment (Table 1).

**Table 1:** Risk factors for shoulder ulcers in sows (modified from Rioja-Lang [2])

Animal-related risk factors	Environment-related risk factors
Body Condition Score (BCS)	Flooring type
Number of litters (parity)	Pen location
Health status	Temperature
Lameness	Humidity
Previous shoulder ulcer history	Type of sow housing
Weaning weight of litter	Friction properties of the floor
Length of lactation period	
Sow behaviour	
Breed	
Genetics	

The three most relevant sow-related risk factors are lameness, BCS and parity [3], among which lameness is the most important [4, 15, 16]. Sows with lameness have a much higher chance of developing shoulder ulcers than do sows with unaffected locomotion [16]. In lame sows, the duration of laying in lateral recumbence during farrowing is increased relative to that in non-lame sows; this laying has been identified as the starting point for the development of shoulder ulcer [11, 12].

In addition to lameness, BCS is closely associated with the development of shoulder ulcers [4–7, 9, 10]. A low BCS increases the likelihood of shoulder ulcer development because of the reduced cushion of fat covering the tuber spina scapulae [2, 17]. Compared to that sows in normal and high body condition,sows with poor body condition have a greater chance of developing shoulder ulcers [16].

Sows that previously developed shoulder ulcers in previous farrowings are at greater risk of developing one in future farrowings. Scar tissue in the skin covering the tuber spina scapulae is indicative of previous shoulder ulcer [7, 11, 12, 18].

### Prevention

Measures to prevent shoulder ulcers in sows include avoiding lameness, maintaining an adequate BCS throughout the previous lactation and recent pregnancy, and the early identification of clinical findings indicative of previous shoulder lesions [2, 13]. Given the broad range of possible causes of lameness, a comprehensive diagnostic approach is required for successful prevention [19]. In particular, hind limb disorders seem to play an important role [15]. Adequate feeding strategies and a proper health status of sows are prerequisites for a sufficient back-fat level. A normal BCS and an adequate back and shoulder fat layer protect against the development of shoulder ulcers [18]. In addition, equipping the farrowing crate floor with a rubber mat for the entire lactation period is associated with a lower frequency of shoulder ulcers [14].

### Treatment

Sows that have recently farrowed should be monitored extensively to ensure that those developing early signs of ulcer development, such as local reddening or swelling, are treated immediately.

For the treatment of shoulder ulcers, local application of a 25% zinc ointment (Apotekets Baby Zinsalve, Denmark) and decompression achieved by a rubber mat (Atlas 18 mm, Kraiburg Elastik GmbH, Tittmoning, Germany) are recommended [20].

In sows suffering from shoulder ulcers that extend to the subcutis or the bone tissue, euthanasia should be considered. Sometimes, early weaning can help stop progression. Pain relief medication can be indicated at a minimum for shoulder ulcers extending to the dermis or the underlying bone [13]. Local antibiotic treatment should be considered for chronic shoulder ulcers as a supportive measure [20].

## Material and methods

### Herd characteristics, sample size and flooring

Data were obtained at a farm with 2300 sows (Landrace x Yorkshire) that employed a one-week batch farrowing system and a suckling period of three weeks. Each week, 105 to 115 sows were farrowing 1400 to 1700 piglets. In the study period the number of weaned piglets per sow was 13.6 and the mean of the number of parities was 2.7. Six groups comprising a total of 659 sows were included in this two-step study.

**Fig. 1:**
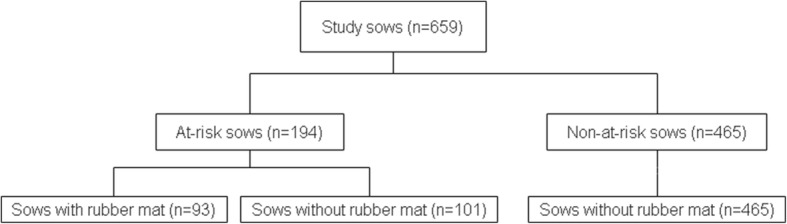
Allocation of sows to the study groups

In the first step, the sows were clinically examined and then allocated to either an at-risk group (*n* = 194) or a not-at-risk group (*n* = 465) according to the examination results. In the second step, the at-risk sows were randomly assigned to a prevention group or a non-prevention group (Fig. 1). The prevention group comprised 93 at-risk sows that were stalled in farrowing crates equipped with rubber mats (PORCA fix D, 65 × 125 cm, 2 cm thickness, Gummiwerk KRAIBURG Elastik GmbH & Co. KG, Tittmoning, Germany). The non-prevention group comprised 101 at-risk sows that were stalled in standard farrowing crates equipped with fully slatted plastic floor fulfilling the minimum requirements of national regulation. The pen size was 250 cm × 180 cm and the crate has a size of 190 cm × 70 cm. Enrichment material was provided by with a metal chain combined with a piece of wood [21].

Sows for which no risk of developing shoulder ulcer was predicted (*n* = 465) were maintained in the same type of standard farrowing pens used for the at-risk sows of the non-prevention group.

### Clinical examination, scoring and image collection

Approximately one week before farrowing, just before the sows were moved from the group housing to the farrowing units, each sow was clinically examined, and the findings were assessed using a clinical scoring system (Tables 2 and 3). Sows with a BCS ≤ 2 (Table 2) and/or locomotion score ≥ 4 (Table 3) and/or scar tissue in the skin covering the spina scapulae were predicted to be at risk for developing shoulder ulcers during the following farrowing period.

**Table 2:** Body condition scoring (modified from Zimmerman [19])

BCS	Condition	Ribs, hip and backbone
1	Excessively thin	Easily visible and palpable
2	Moderately thin	Can be palpated with slight pressure
3	Ideal	Can be palpated with firm pressure; cannot be observed visually
4	Moderately fat	Cannot be palpated
5	Excessively fat	Cannot be palpated

**Table 3:** Semi-quantitative locomotion scoring

Score	Criteria
0	No clinical signs of lameness
1	Barely visibly lame
2	Visibly, moderately lame with pressure on all limbs
3	Visibly lame with decreased pressure on the limbs
4	Decreased limb pressure to achieve relief or avoidance of pressure, or adoption of relieving posture
5	Avoidance of any limb pressure, exclusive adoption of relieving posture

### Longitudinal shoulder scoring during the lactation period

The sows were clinically examined for the status-quo analysis before farrowing and monitored over the three weeks of the lactation period. During the post-partum (p.p.) monitoring period, each sow was examined five times, between p.p. days 2 to 4, 4 to 6, 6 to 8, 9 to 11 and 16 to 18. The examination intervals differed among the sows because the farrowing period of the group spanned three days.

**Fig. 2:**
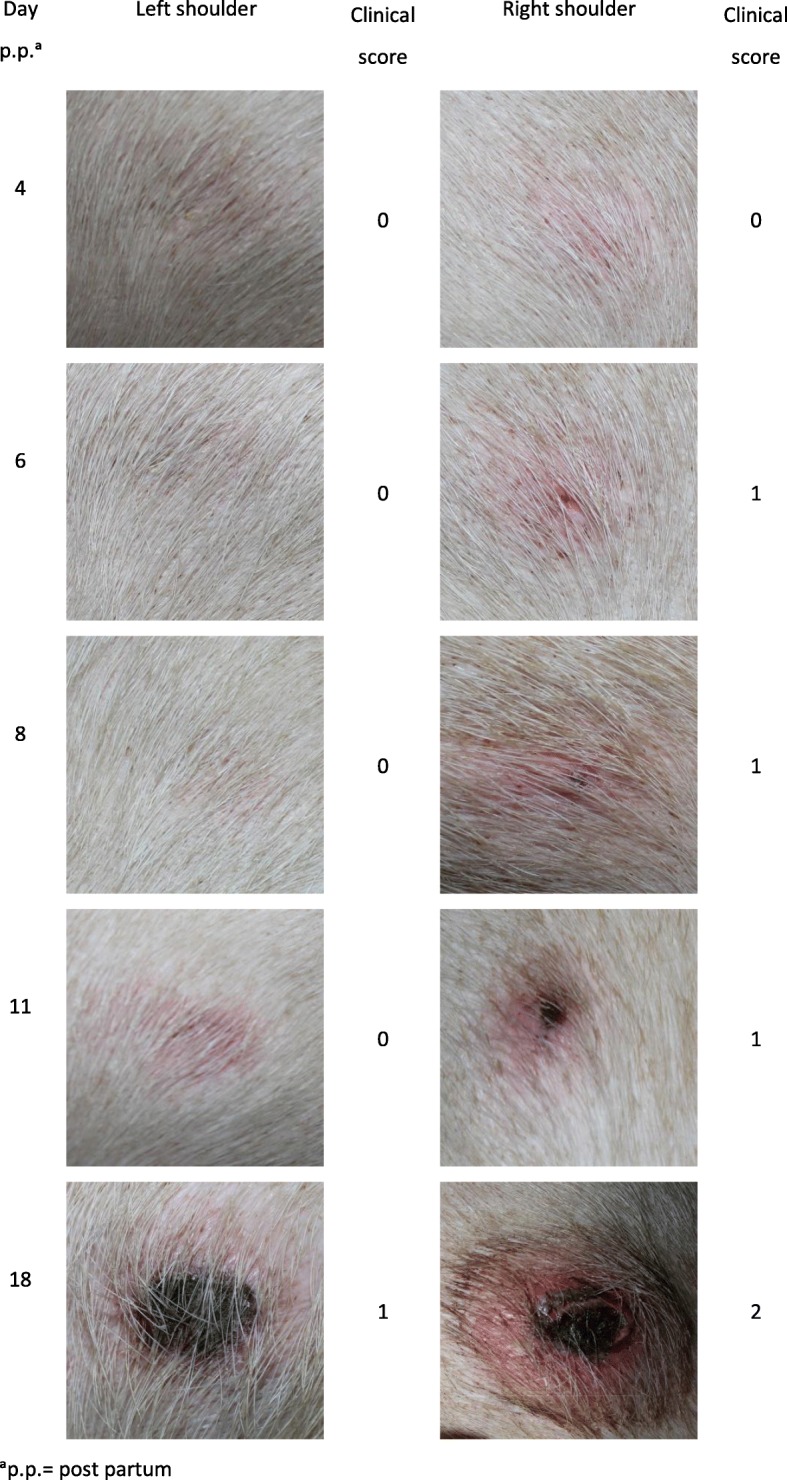
Example photographs of a sow’s shoulders (ID 2861G1) after farrowing

The shoulders were scored according to the clinical findings (Table 4) and photographed (Fig. 2) at each examination time.

**Table 4:** Classification of the clinical findings of shoulder ulcers in sows (Lund [22])

Score	Clinical findings
0	No ulcer; fighting wound
1	Ulceration limited to the epidermis; sometimes covered with a moderate scab
2	Ulcerated dermis, sometimes covered with a scab, usually a small amount of granulation tissue or fibrosis bordering the ulcer
3	Subcutaneous tissue ulcerated, sometimes covered with a scab and accompanied by extensive surrounding granulation tissue or fibrosis
4	Ulceration with exposed bone (tuber spina scapula) accompanied by heavy proliferation of new bone tissue

### Treatment of sows developing shoulder ulcers

For animal welfare reasons, all sows that developed a shoulder ulcer with a score ≥ 2 (see Table 5) were treated regardless of study group. Treatment included Meloxicam (Metacam® 20 mg/ml, Boehringer Ingelheim Vetmedica GmbH, Ingelheim/Rhein, Germany) injection, local application of zinc oxide ointment (Zincojecol WDT® 280 mg/g, Wirtschaftsgesnossenschaft Deutscher Tierärzte eG, Garbsen, Germany) and installation of a rubber mat (PORCA fix D, 65 cm × 125 cm, 2 cm thick, Gummiwerk KRAIBURG Elastik GmbH & Co. KG, Tittmoning, Germany) on the floor of the farrowing crate.

**Table 5:** Maximum expression of clinical shoulder ulcer score throughout day 2 to 18 of the lactation period in the three study groups (frequency; % in parentheses)

	Shoulder ulcer score				
Study group	0	1	2	3	4
Non-at-risk sows	358 (77.0)	97 (20.9)	8 (1.7)^b^	2 (0.4)^b^	0 (0.0)
No-RM^a^ at-risk sows	38 (37.6)	49 (48.5)	11 (10.9)^b^	2 (2.0)^b^	1 (1.0)^b^
RM at-risk sows	49 (52.7)	36 (38.7)	7 (7.5)^b^	1 (1.1)^b^	0 (0.0)

^a^RM = rubber mat

^b^Sows received a treatment with Meloxicam injection, zinc ointment and rubber mat

### Statistical analyses

The primary endpoint was defined as shoulder ulcer yes/no, meaning that if shoulder scores (grade 1 or greater) were present at least one day during the study period, the primary endpoint was set to “yes” else to “no”.

Calculation of the minimum sample size of sows to include was performed by a χ2 test for homogeneity using NCSS-Pass, version 14 [23]. For this calculation, the expected prevalence of shoulder ulcers was determined through a pre-study survey on the farm. The results indicated that a total of 140 at-risk sows were needed to achieve a power greater than 0.8 at a target alpha of 0.05. The calculated minimum sample size was 350 sows.

Logistic regression was used to analyse the primary endpoint of shoulder ulcer (yes/no) Logistic regression was used to analyse the primary endpoint of shoulder ulcer (yes/no) in at-risk vs. non-at-risk sows and in at-risk sows with a rubber mats vs. non-at-risk sows without rubber mats. Odds ratios are reported with 95% confidence intervals. A *p*-value < 0.05 was considered statistically significant. All statistical analyses were performed using the statistical environment R, version 3.5.1 [24].

## Results

Among the 659 sows included in the study, 32.5% developed shoulder ulcers. The prevalence of shoulder ulcers was 23.0% in non-at-risk sows, 62.4% in at-risk sows without a preventive rubber mat (no-RM) and 47.3% in at-risk sows with a preventive rubber mat (RM).

The highest score of shoulder ulcer developed in an individual sow within the examination period was termed the maximum expression (Table 5). In 0.4% of the non-at-risk sows, the maximum expression score was 3, and no sow developed a shoulder ulcer of score 4. In the no-RM at-risk sows, the maximum expression scores were 3, observed in 2.0% of the sows, and 4, observed in 1.0% of the sows. In the prevention group (RM at-risk sows), the maximum expression score was 3, observed in 1.1% of the sows, and no sow developed a shoulder ulcer of score 4.

### Prediction of shoulder ulcers by clinical examination before farrowing

The prediction of the development of shoulder ulcer was analysed by comparing the post-farrowing findings with the prognosis based on the findings of the clinical examination performed pre-farrowing. As half of the at-risk sows had rubber mats installed in their stalls for prevention, only those at-risk sows that did not receive this preventive measure were included in this statistical analysis. A shoulder ulcer was diagnosed in 62.4% of these at-risk sows and in 23.0% of the non-at-risk sows throughout day 2 to 18 of the lactation period. The chance to develop a shoulder ulcer was 5.55 higher for the at-risk sows than for the non-at-risk sows (95% CI: 3.51–8.76, *p* < 0.0001) (Table 6, upper part).

**Table 6:** Association of risk factors with shoulder ulcers

	Presence of shoulder ulcers (any score ≥ 1) *n* (%)	Absence of shoulder ulcers *n* (%)	Odds Ratio (95% Confidence Interval)	*p*-value
Subgroup without rubber mat (*n* = 566)				
At-risk group	63 (62.4)	38 (37.6)	5.55 (3.51, 8.76)	<.0001
Non-at-risk group	107 (23.0)	358 (77.0)	1.0 (Ref.^a^)	
Subgroup under risk (*n* = 194)				
Rubber mat group	44 (47.3)	49 (52.7)	0.54 (0.31, 0.96)	0.0358
No rubber mat group	63 (62.4)	38 (37.6)	1.0 (Ref.^a^)	

^a^Ref. = Reference group

### Effect of a preventive rubber mat installed before farrowing on the development of shoulder ulcers

The effect of a preventive rubber mat on the development of should ulcers in at-risk sows was analysed by comparing the post farrowing findings from at-risk sows between the RM group and the non-RM group. A shoulder ulcer was found in 62.4% of the non-RM at-risk sows and in 47.3% of the RM at-risk sows (Table 6). Among these sows, those with a rubber mat were 0.54 fold less likely to develop a shoulder ulcer than were sows without a rubber mat (95%CI: 1.04–3.27, *p* = 0.0358) (Table 6, lower part).

## Discussion

Shoulder ulcers in breeding sows are an important animal welfare issue. In particular, ulcers extending to the subcutis or the bone tissue of the spina tuber scapulae are substantial animal welfare-related lesions [1]. Clinical studies concerning the prevention of shoulder ulcers have mainly focused on the identification of risk factors, such as body condition score and lameness [2]. The objective of this study was to identify sows at risk of developing shoulder ulcers pre-farrowing and assess how effective risk assessment and the use of rubber mats are for preventing shoulder ulcers in at-risk sows. A preliminary study was conducted beforehand to determine the minimum required sample size for the two-step study. With regard to the maximum expression of shoulder ulcers (Table 5), all sows attaining a shoulder ulcer score of 2 were treated with Meloxicam injection, zinc ointment and provided with a rubber mat regardless of treatment group. This shoulder ulcer treatment may have influenced ulcer progression but not onset.

Analysis of the maximum expression of clinical shoulder ulcers throughout day 2 to 18 of the lactation period showed that 15.0% of the 214 sows that developed ulcers reached an ulcer score ≥ 2. In the non-at-risk group, 2.2% of the sows developed ulcers with a score ≥ 2. In the no-RM at-risk group, 13.9% of the sows developed ulcers with this score, whereas 8.6% of the RM at-risk sows did so. The low number of sows with scores of 3 or 4 (Table 5) is likely the result of the treatment (rubber mat plus daily local application with zinc ointment) of each sow immediately received upon a shoulder ulcer score of 2. The therapeutic effect of this treatment has been demonstrated previously [20].

The data show that reliable prediction of shoulder ulcers can be accomplished by a clinical examination before farrowing, with focus on three clinical parameters. Sows with a BCS ≤ 2 (Table 2) and/or locomotion score ≥ 4 (Table 3) and/or scar tissue in the skin covering the tuber spina scapulae had a 5.55-fold higher odds of developing shoulder ulcers until day 18 of the lactation period relative to sows exhibiting none of these conditions. The risk factors BCS, lameness and the development of a shoulder ulcer in a previous farrowing have been identified in retrospective analyses [4–7, 9]; however, only one previous study used low BSC and presence of scar tissue to predict the risk of shoulder ulcer and applies this information for prevention [25]. According to modern concepts supporting animal health, it is important that not only proven therapeutic measures be applied [20] but also more effective prevention methods be developed. The clinical evaluation of BCS, locomotion and the skin in the area covering the tuber spina scapulae in sows is easily performed by veterinarians as well as veterinarian-trained farmers. It is recommended that the clinical evaluation of these risk factors is performed when the sows are moved from the group house to the farrowing unit.

In those sows found to be at risk for the development of shoulder ulcers, the use of a rubber mat fixed to the floor of the farrowing crate was found to be effective as a preventive measure. At-risk sows that farrowed in crates equipped with rubber mats had a 0.54-fold lower odds of developing shoulder ulcers than did those that farrowed in standard crates. The preventive effect of a rubber mat with a core of foam (centre: 45 mm thick; edge: 17 mm thick) was reported previously for sows with a low BCS or scar tissue in the shoulder area [25]. Core foam rubber mats are likely more decompressive compared to hard core rubber mats. In our study, the use of a solid soft rubber mat (2 cm thick) had a protective effect. The use of rubber mats has been proven effective in the therapy of shoulder ulcers [20] and can be recommended for preventive application.

## Conclusions

The study evaluates the prevention of shoulder ulcer in sows. The pre-farrowing clinical evaluation of BCS, locomotion and the skin in the area over the tuber spina scapulae allowed the prediction of the odds (OR 5.55, *p* < 0.0001) of a sow developing a shoulder ulcer during the subsequent farrowing. By equipping farrowing crates with soft rubber mats as a preventive measure, the odds of an at-risk sow developing a shoulder ulcer was significantly reduced relative to that of an at-risk sow farrowed in a standard crate (OR 0.54, *p* = 0.0358). The study showed that effective prevention of shoulder ulcer can be obtained by simple clinical evaluation of a few risk factors and by equipping the farrowing crate with a soft rubber mat.

## Data Availability

Please contact the corresponding author for data requests.

## Abbreviations

BCS: body condition score
CI: confidence interval
OR: odds ratio
RM: rubber mat
